# WW domain-containing oxidoreductase in neuronal injury and neurological diseases

**DOI:** 10.18632/oncotarget.2961

**Published:** 2014-12-10

**Authors:** Hsin-Tzu Chang, Chan-Chuan Liu, Shur-Tzu Chen, Ye Vone Yap, Nan-Shang Chang, Chun-I Sze

**Affiliations:** ^1^ Department of Cell Biology and Anatomy, College of Medicine, National Cheng Kung University, Tainan, Taiwan; ^2^ Institute of Molecular Medicine, College of Medicine, National Cheng Kung University, Tainan, Taiwan; ^3^ Advanced Optoelectronic Technology Center, National Cheng Kung University, Tainan, Taiwan

**Keywords:** WWOX, WOX1, neurodegeneration, neurites, neuronal death, transcription factors

## Abstract

The human and mouse *WWOX/Wwox* gene encodes a candidate tumor suppressor WW domain-containing oxidoreductase protein. This gene is located on a common fragile site *FRA16D*. WWOX participates in a variety of cellular events and acts as a transducer in the many signal pathways, including TNF, chemotherapeutic drugs, UV irradiation, Wnt, TGF-β, C1q, Hyal-2, sex steroid hormones, and others. While transiently overexpressed WWOX restricts relocation of transcription factors to the nucleus for suppressing cancer survival, physiological relevance of this regard *in vivo* has not been confirmed. Unlike many tumor suppressor genes, mutation of *WWOX* is rare, raising a question whether *WWOX* is a driver for cancer initiation. *WWOX/Wwox* was initially shown to play a crucial role in neural development and in the pathogenesis of Alzheimer's disease and neuronal injury. Later on, *WWOX/Wwox* was shown to participate in the development of epilepsy, mental retardation, and brain developmental defects in mice, rats and humans. Up to date, most of the research and review articles have focused on the involvement of WWOX in cancer. Here, we review the role of WWOX in neural injury and neurological diseases, and provide perspectives for the WWOX-regulated neurodegeneration.

## INTRODUCTION

Human and mouse WWOX/Wwox gene was first cloned in year 2000 [1-5; reviews]. Later on, the mouse *Wwox* genome, which has one million bases, was isolated [6]. WW domain-containing oxidoreductase (designated WWOX, FOR, or WOX1) is a candidate tumor suppressor. This 46-KDa protein has two *N*-terminal WW domains and one *C*-terminal SDR (short-chain alcohol dehydrogenase/reductase), plus a nuclear localization signal located in between the WW domains. Human *WWOX* gene, encoding the WWOX protein, has been mapped to a fragile site on the chromosome ch16q23.3-24.1 [1-5; reviews]. WW domains have been shown to interact with a wide variety of signaling proteins and functioning as adaptor proteins, transcriptional co-activators, and probably ubiquitin ligases. The first WW domain of WWOX binds a broad spectrum of PPxY-containing proteins, including p63, p73, AP-2γ (Activator protein 2γ), ErbB4 (v-Erb-B2 avian erythroblastic leukemia viral oncogene homolog 4), Runx-2 (Runt-related transcription factor 2), Dvl-2 (Dishevelled homolog protein-2), SIMPLE (Small integral membrane protein of the lysosome/late endosome), MET (MET proto-oncogene), LMP2A (viral latent membrane protein 2A), and others [1-13]. In stark contrast, when WWOX becomes Tyr33-phosphorylated in the first WW domain, it acquires an enhanced binding capability with PPxY motif-deficient proteins such as p53, JNK1 (c-Jun *N*-terminal kinase 1), c-Jun (Jun proto-oncogene), CREB (cAMP responsive element binding protein), and Zfra (Zinc finger-like protein that regulates apoptosis) [4,8,10-14].

Additionally, the *C*-terminal SDR domain of WWOX physically interacts with membrane hyaluronidase Hyal-2 [15], tau [16] and GSK-3β (Glycogen synthase kinase 3 beta) [17]. We have shown that WWOX is an inhibitor of neurodegeneration, because of its interaction with tau and inhibition of enzyme-dependent Tau hyperphosphorylation [16,17]. WWOX is involved in the TGF-β (Transforming growth factor beta) signaling [15]. Hyal-2 binds TGF-β1 and acts as a receptor. During signaling, Hyal-2 is internalized and recruits WWOX via binding to the SDR domain, and the resulting WWOX/Hyal-2 complex binds Smad4 and is then accumulated in the nucleus, which affects cell survival or death [15]. WWOX physically interacts with MEK1 (Mitogen-Activated Protein Kinase Kinase 1), and that dissociation of the protein complex results in apoptosis of leukemia cells [18]. Förster resonance energy transfer (FRET) analysis revealed that both the *N*-terminal WW domain and the *C*-terminal SDR domain are capable of interacting with MEK1 [18].

Supporting evidence from *Drosophila* and mouse knockout models has revealed that WWOX acts more than just a tumor suppressor [7-12]. Overexpression of the full-length WWOX or its WW or SDR domain region induces apoptosis [4,8,19]. WWOX enhances tumor necrosis factor (TNF) cytotoxicity by down-regulation of the apoptosis inhibitors Bcl-2 and Bcl-xL. Under stress conditions, activated or Tyr33-phsphorylated WWOX binds p53, in which the complex co-translocates to the mitochondria or to the nucleus [4,8,20]. WWOX in the cellular or nuclear compartment may interfere with genes transcription or cancers response to chemotherapy [13,21].

WWOX is involved in binding and regulating GSK-3β activity, and this limits Tau hyperphosphorylation, neurite outgrowth in neuronal differentiation, and formation of neurofibrillary tangles (NFTs) and senile plaques in Alzheimer's disease (AD) [16,17,22]. Neural injuries to the brain, spinal cord, or peripheral nerve are devastating, which often leads to complex and lifelong disability. These injuries could be acute or chronic and continuously affect the remaining undamaged nervous system. Neural injuries cause damage to the neurons, its processes or neurites, and neural supporting cell or glial cells. Data collect from Genome Wide Association Studies (GWAS) and full knock-out (KO) mice models have implicated that *WWOX* gene may be associated with metabolic syndrome and related conditions that affecting cardiovascular and neurological systems [23-27]. Given WWOX interacts with molecules involved in cell signaling, gene transcription, and lipid metabolism, all of which may regulate cell survival or death. It is very likely that WWOX plays a critical role in central nervous system (CNS) physiology and injury. In this article, we review the possible perspectives that WWOX may be involved in neural injury and its potential role in the pathogenesis of neurological diseases.

### WWOX in neuronal death signaling

**Figure 1:**
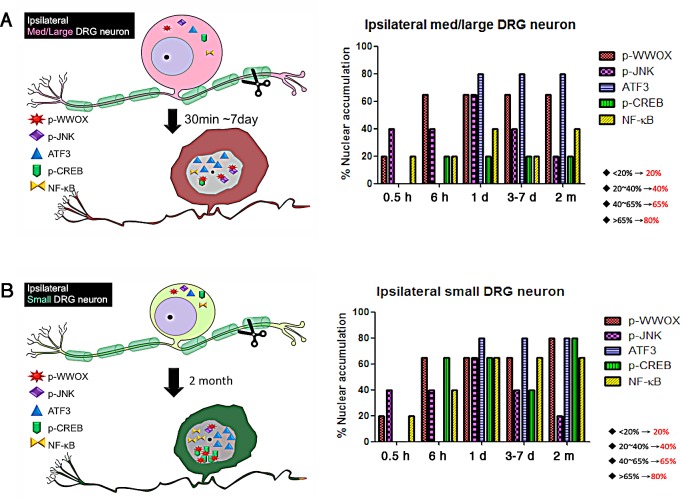
Relocation of WWOX and transcription factors to the nuclei upon neuronal injury (A) Rats were subjected to sciatic nerve axotomy. During the acute phase of injury, activated p-JNK and p-WWOX rapidly relocated to the nuclei of injured neurons in 30 min in the ipsilateral side, followed by continual accumulation of activated transcription factors CREB, NF-κB, ATF3 and others to in the nuclei of injured medium/large-size neurons in 1 to 7 days. JNK and CREB are likely to block the proapoptotic function of WWOX via direct binding [31]. Neuronal death can occur in 7 days. (B) In the chronic phase, gradual accumulation of activated JNK, WWOX, CREB, ATF3 and NF-κB in the nuclei was found in the small neurons post injury for 2 months. The schematic graphs were drawn based on published data [31]. The percentages of protein nuclear accumulation are shown. The actual range of each bar is shown in the lower right.

Cell death occurs in neural injury or neurodegenerative diseases. The level of TNF receptor I (TNFR1) is up-regulated in AD, which correlates with the apoptotic process through its ligand TNF-α (tumor necrosis factor alpha) [28-30]. TNF-α induces inflammatory response and apoptosis by activating TRADD (TNF receptor-associated protein with death domain), FADD (Fas-associated protein with death domain), JNK1, WWOX, and NF-κB (nuclear factor-kappa B) [1,2,28-30]. TNF-α induces activation of JNK1 in AD patients and mouse models of AD, as evidenced by the expression of pro-apoptotic genes and activation of caspases-3 and caspase-9 [28-30]. Additionally, sciatic nerve transection could lead to neuronal injury and death. This effect rapidly results in activation of JNK1 and WWOX as short as 30 min in the injured DRG neurons in rats. Subsequently, there are significantly increased accumulation of WWOX, JNK1, CREB, c-Jun, NF-κB and ATF3 in the nuclei of injured large neurons within 24 hours or during the first week of the injury [31] (Figure 1). Later, in the chronic phase of the neuronal injury, concurrent activation of WWOX, CREB, and NF-κB occurs in small neurons prior to apoptosis [31]. WWOX strongly binds CREB in the nuclei. Additionally, WWOX blocks the promoter activation governed by the prosurvival CREB, CRE and AP-1 *in vitro*. In contrast, WWOX enhances the promoter activation regulated by c-Jun, Elk-1 and NF-κB [31]. Whether WWOX regulates the function of transcription factors in neuronal survival or death *in vivo* remains to be established.

WWOX interacts with p53 and both proteins act synergistically to induce apoptosis [4,8,10,14,19]. Intriguingly, soluble Aβ (amyloid beta) peptides are involved in HIPK2 (Homeodomain-interacting protein kinase 2) degradation, which results in misfolded p53 and altered vulnerability of cells to noxious stimulus, suggesting that conformational changed p53 can be a putative biomarker for AD [32,33]. Whether or not WWOX interacts with Aβ peptides or HIPK2 remains to be established; however, it is important to discern the link between transient overexpression of WWOX or SDR domain and accumulation of Aβ peptide, Tau phosphorylation, and formation of NFTs.

### WWOX in neurological disease pathology

Tau is a microtubule-associated protein functioning to promote microtubule assembly and is essential for the axonal transportation. Phosphorylation of Tau affects axonal flow and cell viability during differentiation [32]. Tau phosphorylation can be regulated by WWOX via its interaction with GSK-3β, JNK1, ERK, Wnt/beta-catenin and Tau [14,16-18,33-36]. GSK-3β maintains a hyperactive state and hyperphosphorylates Tau in AD. GSK-3β regulates APP (amyloid precursor protein) metabolism and overproduction of Aβ that leads to reduced neurogenesis and increased apoptosis [37]. Protein phosphatase 2A (PP2A) is shown to be a key enzyme in dephosphorylating Tau [38].

WWOX binds directly to Tau through its SDR domain. Silencing of WWOX by small interfering RNA increases the binding of Tau to GSK-3β and Tau phosphorylation, indicating that WWOX is involved in regulating GSK-3β activity [16]. Overexpression of WWOX enhances the SH-SY5Y cell differentiation with or without the treatment of retinoic acid (RA). In contrast, knockdown of WWOX in RA-differentiated SH-SY5Y cells causes a decrease in neurite outgrowth, suggesting a role of WWOX in neuronal differentiation [17]. The physical interactions of WWOX with Tau, JNK1 and GSK-3β have been demonstrated in the rat brains extract and cultured cells [16,17]. These findings suggest that WWOX may participate in AD pathology through its protein-binding partners.

**Figure 2:**
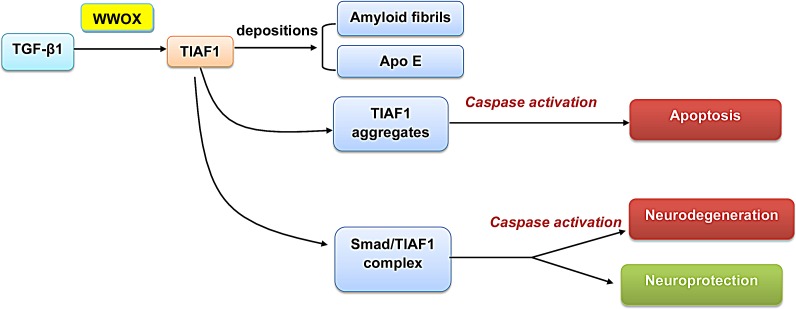
A schematic graph of TGF-β1-regulated neurodegeneration Both WWOX and TIAF1 participate in regulating apoptosis and neurodegeneration [15,40]. Aggregated TIAF1 induces apoptosis in a caspase-dependent manner, whereas Smad4 controls TIAF1 aggregation [40]. Long-term TGF-β1 exposure may result in irreversible formation of amyloid fibrils and apolipoprotein E (ApoE) depositions *in vivo* [39].

WW domain-containing proteins participate in the TGF-β signaling [15]. TGF-β1 is crucial in regulating neuroprotection and neurodegeneration [39-41] (Figure 2). Recently, we have identified that a small TGF-β1-induced antiapoptotic factor (TIAF1) is involved in the pathogenesis of AD [40]. Long-term TGF-β1 exposure results in irreversible formation of amyloid fibrils and apolipoprotein E (ApoE) depositions *in vivo*, even after silencing of the transgene or under TGF-β1 removal [39]. Both WWOX and TIAF1 participate in regulating the activation of Smad-driven promoter via type II TGF-β1 receptor (TβRII)-independent manner to induce apoptosis or neurodegeneration [15,40]. The differences of TGF-β1 in exerting neuroprotection or degeneration may be related to the TIAF1/Smad4 complex formation, as Smad4 limits the polymerization of TIAF1 [40]. WWOX is able to interact with TIAF1 (Chang et al., unpublished), whereas whether WWOX prevents TIAF1 aggregation is unknown. Aggregated TIAF1 induces apoptosis in a caspase-dependent manner. Under physiological conditions, TGF-β1 signals the binding of TIAF1 with Smad4 to form a complex, which relocates to the nuclei to modulate gene transcription [40]. Smad proteins are involved in transcribing the gene coding for membrane APP [41,42]. These studies further dissect the role of WWOX in TGF-β1-induced TIAF1 self-aggregation and Smad4 overexpression in senile plaques formation, which might shed light for the development of therapeutic strategy in neurodegenerative diseases.

### WWOX in metabolic syndrome and neural development

**Figure 3:**
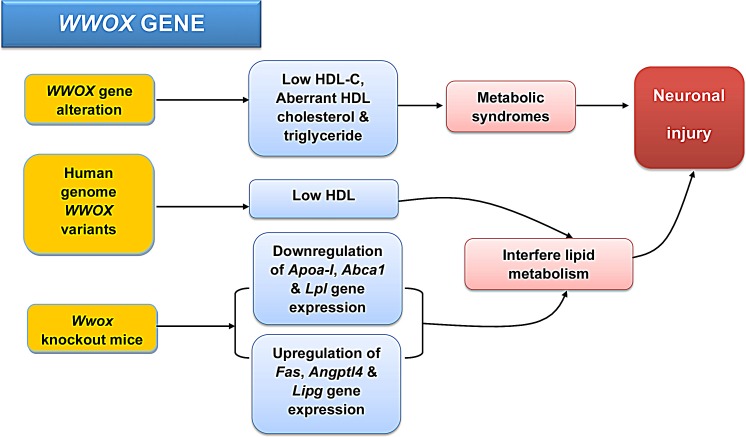
*WWOX* in metabolism and metabolic diseases *WWOX* gene alteration is associated with low plasma HDL-C levels and aberrant HDL cholesterol and triglyceride levels, and these may lead to the development of metabolic syndromes and chances of neuronal injury [45]. Eight variants have been identified in the human *WWOX* genome, which are significantly associated with the low-HDL trait [47]. In *Wwox* knockout mice, the expression levels of several genes and proteins are altered [47].

Complications from metabolic syndromes such as high blood cholesterol, hypertension, obesity, and diabetes interfere blood perfusion and energy supply to the nervous system and increase the risk of neuronal injury. Genome based gene analyses have identified *WWOX* as a hypertension (HTN) susceptibility gene in Asian populations [43,44]. HTN candidate genes or HTN itself in humans and mice are associated with obesity, glucose metabolism, ion homeostasis, diabetic mellitus, and cardiovascular or neurological dysfunctions. All of which are important for the development of metabolic syndromes. In addition, *WWOX* gene alteration is associated with low plasma HDL-C levels and aberrant HDL cholesterol and triglyceride levels, which are crucial for the development of metabolic syndromes and increases in the risk of neuronal injury [45] (Figure 3). Multiple metabolic defects occur in whole body and conditional *Wwox* knockout mice, further supporting *Wwox* gene as a key regulator in different metabolic processes [45,46]. Additionally, eight variants have been identified in the human *WWOX* genome, which are significantly associated with the low HDL trait in two multi-generational French Canadian dyslipidemic families [47] (Figure 3). Similarly, in whole body and liver conditional *Wwox* knockout mice, there are decreased protein levels of ApoA-I (apolipoprotein A-I) and ABCA1 (ATP-binding cassette transporter) levels in hepatic tissues, along with reduction in the mRNA expression of *Apoa-I* and *Lpl (lipoprotein lipase)*, upregulation in *Fas*, *Angptl4* (angiopoietin-like 4) and *Lipg* (endothelial lipase) [47] (Figure 3). These observations suggest a significant role for WWOX in modulating HDL and lipid metabolism, including cholesterol homeostasis, ApoA-I/ABCA1 pathway, and fatty acid biosynthesis/triglyceride metabolism [47]. Interference in lipid metabolism may be a critical contributor in the pathogenesis of neurological diseases.

Large consanguineous family studies have reported that homozygous *WWOX* point mutations (e.g. P47T and G372R) could lead to a new form of childhood onset autosomal recessive cerebellar ataxia and epilepsy [26]. A recent case showed that homozygous nonsense mutation of *WWOX* results in protein loss of function, and the patient suffers from severe anomalies, including short and growth retardation, microcephaly with seizure, retinal degeneration, and early death at 16 months of age [27]. In parallel, 13-bp deletion in exon 9 of *Wwox gene* has been found in *Ide/Ide* rat and this results in a frameshift reading of *WWOX* codon at the *C*-terminus [48]. Nonetheless, WWOX protein is barely detectable in the testes and hippocampi of *lde/lde* rats, suggesting that the *C*-terminus of WWOX is critical to protein stability. In a striking similarity to those symptoms in humans, the *lde/lde* rats are shown to have dwarfism, postnatal lethality, male hypogonadism, and a high incidence of epilepsy and many vacuoles in the hippocampus and amygdala [48]. Nonetheless, despite the loss of function of WWOX protein, no tumor formation was shown in the aforementioned cases [26,27,48]. Overall, these reports clearly indicate that germline loss-of-function of *WWOX* leads to developmental deficiency in the neural system.

In contrast to the aforementioned observations, functional deficiency of *WWOX* in *Drosophila* fails to generate the disease phenotypes in the neural system [49]. It has been suggested that WWOX functions in aerobic glycolysis metabolism (Warburg effect) and regulates reactive oxygen species [49]. WWOX is functionally linked to either CG6439/isocitrate dehydrogenase (*IDH*) or Cu-Zn superoxide dismutase (*SOD*), whereas direct binding interactions among these proteins are unknown. These proteins may co-localize and function together in the mitochondria [1,2,4]. Oxidative phosphorylation increases a steady-state transcription of *WWOX* gene, whereas glycolysis downregulates the expression [50]. The observation further supports the essential role of WWOX in the mitochondria, and that downregulation of WWOX in AD causes neuronal damage [1,2,4].

**Figure 4:**
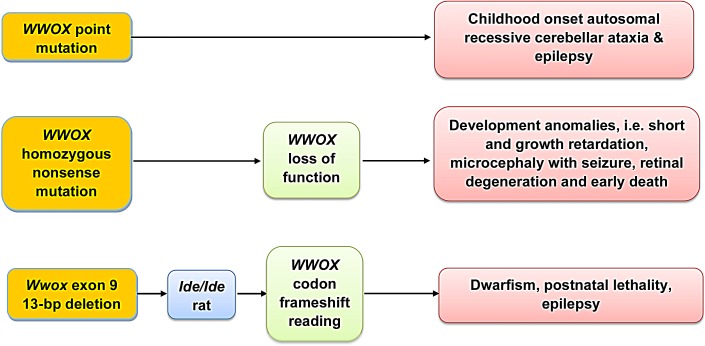
*WWOX* gene mutations Homozygous *WWOX* point mutations (e.g. P47T and G372R) could lead to a new form of childhood onset autosomal recessive cerebellar ataxia and epilepsy [26]. Also, homozygous nonsense mutation of *WWOX* causes protein loss of function, and the patient suffers from severe anomalies, including short and growth retardation, microcephaly with seizure, retinal degeneration, and early death at 16 months of age [27]. A 13-bp deletion in exon 9 of *Wwox gene* is found in *Ide/Ide* rat and this results in a frameshift reading of *WWOX* codons at the *C*-terminus [48].

## CONCLUSION AND PERSPECTIVES

In conclusion, substantial evidence has shown that WWOX participates in the control of the function of transcription factors *in vivo*. For example, WWOX could significantly promote the NF-κB-induced promoter activation [31]. WWOX also regulates the transcriptional activation of CREB, CRE, c-Jun, Elk-1 and AP-1 [31]. Apparently, under normal physiology, this regulatory control is likely to affect neuronal degeneration or regeneration and cell metabolism. *WWOX*/*Wwox* is involved in the maintenance of lipid metabolism [26,27,45-48]. Alterations of *WWOX*/*Wwox* gene, including point mutation, missense or nonsense mutation, and deletion, may lead to ataxia, epilepsy, dementia, neurodegeneration, and diseases associated with HDL lipid metabolism [16,17,26,27,45-48]. Although overexpressed *WWOX*/*Wwox* may induce death of many types of cancer cells [1-11], no spontaneous cancer formation occurs in humans with nonsense mutation of this gene. Further, knockdown of *WWOX*/*Wwox* may readily induce apoptosis of many types of normal and cancer cells. Conceivably, WWOX plays a crucial role in cell survival by controlling metabolism. We have shown that the *C*-terminal SDR domain of WWOX is responsive to stimulation by sex steroid hormones androgen and estrogen, suggesting that WWOX may act as a hormone receptor [51,52]. To play this role, a portion of WWOX is located in the mitochondria to carry out its oxidoreductase function. Functional alteration of this event would cause the aforementioned metabolic diseases. Finally, p53 and WWOX are partners in inducing apoptosis [20]. In the absence of WWOX, p53 tends to become destabilized and subjected to degradation [20]. p53 is involved not only in tumor suppression but also in aging process and metabolic events [53,54]. Conceivably, the partnership between p53 and WWOX is crucial in the aging event and metabolic regulations.
